# *L1* and *L2* gene polymorphisms in HPV-58 and HPV-33: implications for vaccine design and diagnosis

**DOI:** 10.1186/s12985-016-0629-9

**Published:** 2016-10-07

**Authors:** Zuyi Chen, Yaling Jing, Qiang Wen, Xianping Ding, Shun Zhang, Tao Wang, Yiwen Zhang, Jianhui Zhang

**Affiliations:** 1 Key Laboratory of Bio-Resources and Eco-Environment, Ministry of Education, Sichuan University, Chengdu, China; 2 Bio-resource Research and Utilization Joint Key Laboratory of Sichuan and Chongqing, Sichuan and Chongqing, China; 3 Institute of Medical Genetics, College of Life Science, Sichuan University, Chengdu, 610064 China

**Keywords:** HPV-58, HPV-33, L1 and L2, Genetic diversity

## Abstract

**Background:**

Cervical cancer is associated with infection by certain subtypes of human papillomavirus (HPV). The *L1* protein comprising HPV vaccine formulations elicits high-titre neutralizing antibodies and confers protection against specific HPV subtypes. HPV *L2* protein is an attractive candidate for cross-protective vaccines. HPV-33 and HPV-58 are very prevalent among Chinese women.

**Methods:**

To study the gene intratypic variations and polymorphisms of HPV-33 and HPV-58 *L1/L2* in Sichuan China, HPV-33 and HPV-58 *L1* and *L2* genes were sequenced and compared with other genes submitted to GenBank. Phylogenetic trees were constructed by maximum-likelihood and the Kimura 2-parameters methods (MEGA 6). The secondary structure was analyzed by PSIPred software, and HPV-33 and HPV-58 L1 homology models were created by SWISS-MODEL software. The selection pressures acting on the *L1/L2* genes were estimated by PAML 4.8.

**Results:**

Among 124 HPV-33 *L1* sequences 20 single nucleotide mutations were observed included 8/20 non-synonymous and 12/20 synonymous mutations. The 101 HPV-33 *L2* sequences included 12 single nucleotide mutations comprising 7/12 non-synonymous and 5/12 synonymous mutations. The 223 HPV-58 *L1* sequences included 32 single nucleotide mutations comprising 9/32 non-synonymous and 23/32 synonymous mutations. The 201 HPV-58 *L2* sequences comprised 26 single nucleotide mutations including 9/26 non-synonymous and 17/26 synonymous mutations. Selective pressure analysis showed that most of the common non-synonymous mutations showed a positive selection. HPV-33 and HPV-58 *L2* were more stable than HPV-33 and HPV-58 *L1*.

**Conclusions:**

HPV-33 and HPV-58 *L2* were better candidates as clinical diagnostic targets compared with HPV-33 and HPV-58 *L1*. Clinical diagnostic probes and second-generation polyvalent vaccines should be designed on the basis of the unique sequence of HPV-33 and 58 *L1/L2* variations in Sichuan, to improve the accuracy of clinical detection and the protective efficiency of vaccines.

**Electronic supplementary material:**

The online version of this article (doi:10.1186/s12985-016-0629-9) contains supplementary material, which is available to authorized users.

## Background

Human papillomavirus (HPV) infection plays a critical role in the development of cervical cancer [1]. The risk of developing cervical cancer in HPV-infected patients is 50-fold higher than in uninfected women [1, 2]. Approximately 500000 new cases of cervical cancer are diagnosed every year, with 250000 deaths, more than 85 % of all patients belong to low-income countries [3, 4].

Genital HPV subtypes are typically divided into two groups according to their presumed oncogenic potential. High-risk oncogenic HPV subtypes −16, −18, −58, −33, −52 and −45 are more common in Asia than elsewhere [5]. HPV-33 and HPV-58 are prevalent among Chinese women, only after HPV-16 [6–8]. HPV-33 and HPV-58 account for approximately 5 and 2 % of cervical cancer cases world-wide [9, 10]. Nevertheless, the carcinoma in situ and cervical cancer cases caused by HPV-58 and HPV-33 outnumber HPV-18, ranking second and third in China, respectively [6, 11]. Compared with the high-risk HPV-18, vaccine design in China is focused predominantly on high-risk subtypes HPV-58, and HPV-33.

The HPV genome is packaged within the major capsid late protein *L1* and the minor capsid proteins L2 [12, 13]. Five *L1* proteins form a pentamer, and 72 pentamers constitute the virus capsid. The *L1* and *L2* proteins self-assemble into virus-like particles (VLPs) that induce high levels of neutralizing antibodies and are highly protective [14, 15]. The L1-VLPs are the components used in the design of specific prophylactic vaccines. Vaccine targeting *L1* only prevents infection by specific HPV subtypes because of the lack of cross-protective epitopes in different HPV subtypes. HPV *L2* protein also induces neutralizing antibodies, the N-terminal of *L2* protein contains cross-protective epitopes and represents the target of neutralizing antibodies [14]. Therefore, targeting *L2* may be an attractive approach for a candidate vaccine.

Vaccines against HPV-16, HPV-18, HPV-6 and HPV-11 *L1* are available [15]. The two HPV vaccines Gardasil and Cervarix target only two high-risk HPV subtypes. Prevention of more than 90 % of HPV infections requires targeting at least 5 additional high-risk HPV subtypes HPV-31, HPV-33, HPV-45, HPV-52 and HPV-58 [16]. Vaccines targeting *L2* and other subtypes of *L1* are currently under investigation [14].

The data supporting HPV-58 *L2* and HPV-33 *L1/L2* in China are limited. The molecular variants of HPV-33 *L1/L2* worldwide are not widely reported. Ethno-geographical variations are observed in distribution of HPV subtypes. Among different subtypes of HPV, there are subtypes and variants that can acquire biological advantages through fixed mutations in their genomes, and even small variations could result in small adaptive improvements that could alter the composition of an HPV-infected population [17]. Altered amino acid composition affects the host immune response, and in such cases, intra-type protection may be less effective [18]. Ideally, the diagnosis and treatment of vaccine constructs needs to be developed locally.

This study investigated the HPV-33 and HPV-58 *L1/L2* gene polymorphism and intratypic variations in Sichuan, China. This study can provide essential data for future research on viral prevention and therapeutics. Above all, our study provides critical data facilitating the development of diagnostic probes and design of vaccines based on HPV-33 and HPV-58 *L1/L2*.

## Methods

### Study population and specimen collection

Cervical specimens were collected from Sichuan Reproductive Health Research Center Affiliated Hospital, The Angel Women’s and Children’s Hospital, The Chengdu Western Hospital Maternity Unit, and The Peoples’ Hospital of Pengzhou, and other institutions. Between January 1, 2009, and December 31, 2014, women presenting for cervical screening underwent histology and cytology evaluations for cervical disease. Women over 14 years of age and with visible cervical lesions and/or HPV-related diseases (e.g., cervicitis, cervical intraepithelial neoplasia) were eligible for inclusion. Cervical specimens were collected from participants and placed in a preservative buffer and stored at −20 °C.

### Genomic DNA extraction

HPV-DNA was extracted and examined using a Human Papillomavirus Genotyping Kit (Yaneng Bio, Shenzhen, China) according to the manufacturer’s instructions. This kit enabled the classification of 23 HPV subtypes (18 types of high-risk HPV 16, 18, 31, 33, 35, 39, 45, 51, 52, 53, 56, 58, 59, 66, 68, 73, 83, MM4 and five types of low-risk HPV genotypes 6, 11, 42, 43, and 44). In total, 478 HPV-58 and 273 HPV-33 positive samples were collected.

### PCR amplification and sequencing

The entire *L1/L2* genes of HPV-33 and HPV-58 were amplified using primer pairs. The primers were designed according to the GeneBank reference sequences for HPV-33 (GenBank: M12732.1) and HPV-58 (GenBank: D90400). The primer sequences were listed in Table 1. Each 50 μL PCR reaction contained 5 μL of extracted DNA (10–100 ng), 200 μmoL MgCl2 and dNTPs, 2 U of Pfu DNA polymerase (Sangon Biotech, Shanghai, China), and 0.25 μmoL of each primer. The PCR conditions were 95 °C for 10 min; 35 cycles of 50 s each at 94 °C, 54 °C (difference for each gene) for 60 s, 72 °C for 60 s, and a final step of 72 °C for 7 min. The PCR amplification products were visualized on 2 % agarose gels stained with GeneGreen nucleic acid dye under the ultra violet light WFH-202. Target products were sequenced by Sangon Biotech.

**Table 1:** Primers used for the molecular characterization of HPV-33 and HPV-58 L1 and L2

Gene	Primer name	Sequence 5′ to 3′	Primer position	Product size (bp)	Annealing Temperature
33 L1	HPV33 L1 1 F	ATTTGTTCCTATTTCGCCT	5468	921	54 °C
	HPV33 L1 1R	CAGCCCTATTAAAAAAGTGTC	6388		
	HPV33 L1 2 F	CAGTACATGCAAATATCCAGATTA	6271	913	54 °C
	HPV33 L1 2R	ACATACACAAAAAACAAACAACA	7183		
33 L2	HPV33 L2 1 F	GCACATGGTGGTGTTTTAAC	4116	935	54 °C
	HPV33 L2 1R	AGTCAGGATCAGGAGCAGGT	5050		
	HPV33 L2 2 F	AATGTAACATCAAGCACGCC	4834	994	55 °C
	HPV33 L2 2R	TAAACGGACCCTAAAAACCC	5827		
58 L1	HPV58 L1 1 F	TCCATTTATTCCTATATCTCCACTA	5515	876	56 °C
	HPV58 L1 1R	GAAAAAAGAACAAACTATCCCCA	6390		
	HPV58 L1 2 F	ATGGTAGATACAGGGTTTGGAT	6240	1044	55 °C
	HPV58 L1 2R	AAACAGGAAACTGACAAAGACA	7283		
58 L2	HPV58 L2 1 F	GTATACTGGTTATGCACATGGT	4125	847	54 °C
	HPV58 L2 1R	AAAAAAGCAGGGTCAACAAC	4971		
	HPV58 L2 2 F	AATGGATACCTTTGTTATTTCTAC	4834	992	54 °C
	HPV58 L2 2R	TACTTTTTTATTGTTATTGGGACT	5825		

### Variant identification and analysis

The sequences and variations were analyzed by NCBI Blast, and DNAMAN version 5.2.2. The nucleotide positions were numbered according to the GeneBank reference sequences of HPV-33 (GenBank: M12732.1) and HPV-58 (GenBank: D90400). All the data were confirmed by repeating PCR amplification and sequence analysis at least twice.

### Phylogenetic trees analysis

Then, phylogenetic trees of respective HPV-33 *L1/L2* and HPV-58 *L1/L2* variation patterns were constructed with the maximum-likelihood trees using MEGA (Molecular Evolutionary Genetics Analysis Version) 6 software and Kimura’s two-parameter model. The tree topologies were evaluated using bootstrap resampled 1,000 times [19].

### Analysis of the selection pressures and secondary structure

To estimate the positive selections at particular sites of the HPV-33 and HPV-58 *L1/L2* gene sequences, the *codeml* program in the PAML (Phylogenetic Analyses by Maximun Likelihood) version 4.8 package was used to perform the likelihood ratio tests (LRTs) to infer non-synonymous and synonymous nucleotide divergence for coding regions by the method of Nei and Gojobor [20–23]. The secondary structure of the reference sequences were analyzed by PSIPred servers at (http://bioinf.cs.ucl.ac.uk/psipred). PSIPred is a simple and accurate secondary structure prediction method, incorporating two feed-forward neural networks, which enable the analysis of output obtained from PSI-BLAST (Position Specific Iterated-BLAST). A very stringent cross validation of the method indicated that PSIPred 3.2 attained an average Q3 score of 81.6 % [24].

### Homology models analysis

HPV33 and HPV58 L1 homology models were created by SWISS-MODEL (http://swissmodel.expasy.org/) based on the crystal structure of the HPV16 L1 pentamer [25, 26], and then visualized using Swiss-PDP viewer V4.0 software.

## Results

Of all the HPV-58 and HPV-33 samples, only 223 sequences of HPV-58 *L1* gene, 201 sequences of the HPV-58 *L2* gene, 124 sequences of the HPV-33 *L1* gene, and 101 sequences of the HPV-33 *L2* gene were obtained owing to the small number of copies of infected HPV in some women and limited amplicons obtained for sequencing, and there maybe a potential sampling bias against integrated HPV genomes resulting in lost capsid genes.

### Gene polymorphism of HPV-33 L1

**Fig. 1:**
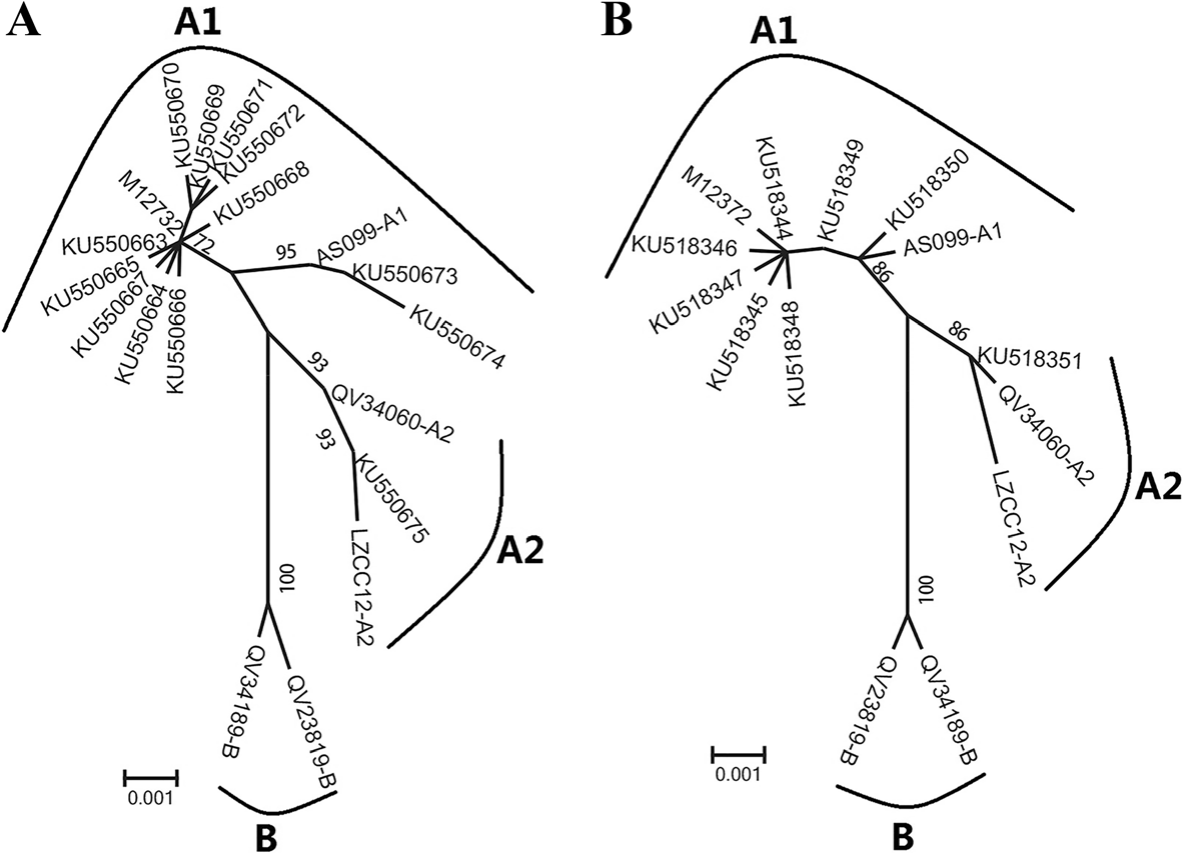
Maximum-likelihood trees of HPV-33 variants. *Note*: Phylogenetic trees based on the Maximum-likelihood method of representing the relationships between variants of HPV-33, (**a**) Tree based on gene *L1*, (**b**) Tree based on gene *L2*. Sub-lineage classification based on full genomes from a reported research [27], and one or two representative sequences were chosen from each branch of the tree, respectively (AS099-A1/HQ537697, INCC0137-A2/HQ537699, QV34060-A2/HQ537698, QV23819-B/HQ537705, QV34189-B/HQ537707). Numbers above the branches indicate the bootstrap values that are greater than 70 %

Compared with the HPV-33 reference sequence (GenBank: M12732.1), the nucleotide variation rate of HPV-33 *L1* was 68.55 % (85/124) in the 124 HPV-33 *L1* sequences studied. We identified 20 single nucleotide changes among the 124 sequences studied. Specifically, 12/20 (60.00 %) were synonymous and 8/20 (40.00 %) were non-synonymous mutations. Only 1 non-synonymous mutation was observed in sequence encoding the helix. The detected mutations are summarized in Table 2. The maximum-likelihood phylogenetic tree can be seen in Fig. 1a. The secondary structure predicting result of the HPV-33 *L1* was showed in Additional file 1: Figure S1 (A).

**Table 2:** Nucleotide sequence mutation at *L1* of 13 HPV-33 isolates

Sequence pattern	HPV33 L1 Nucleotide sequence																				*n*	Sub-lineages
	5	5	5	5	6	6	6	6	6	6	6	6	6	6	6	6	6	6	7	7		
	7	9	9	9	3	3	3	4	4	4	5	5	6	6	6	6	7	9	0	0		
	6	6	9	9	8	9	9	6	7	8	2	4	1	6	7	9	4	5	4	6		
	0	0	0	7	5	0	6	3	8	7	0	4	3	4	3	4	8	1	4	3		
M12732	C	T	G	A	T	C	G	T	T	A	G	A	T	A	A	A	A	A	C	G		
KU550663	-	-	-	-	-	-	-	-	-	-	-	-	-	-	-	-	-	-	-	-	39	A1
KU550664	-	C	-	-	-	-	-	-	-	-	-	-	-	-	-	-	-	-	-	-	4	A1
KU550665	-	-	-	-	-	-	-	-	-	-	-	-	-	-	-	-	-	C	-	-	1	A1
KU550666	-	-	-	-	-	-	-	-	-	-	-	-	-	-	-	G	-	-	-	-	1	A1
KU550667	-	-	-	-	-	-	-	-	-	-	-	-	-	-	-	-	-	-	A	-	2	A1
KU550668	-	-	-	-	-	-	-	-	-	-	-	-	-	-	-	-	-	-	-	A	54	A1
KU550669	-	-	-	-	-	-	-	-	-	-	-	-	C	-	-	-	-	-	-	A	1	A1
KU550670	-	-	-	-	-	-	-	-	-	-	A	-	-	-	-	-	-	-	-	A	2	A1
KU550671	-	-	-	-	G	-	-	-	-	-	-	-	-	-	-	-	-	-	-	A	1	A1
KU550672	-	-	-	-	-	-	-	-	-	C	-	-	-	-	-	-	-	-	-	-	1	A1
KU550673	-	-	-	G	-	A	A	-	-	-	-	G	-	G	-	-	-	-	-	-	4	A1
KU550674	-	-	-	-	-	A	A	C	-	-	-	G	-	G	-	-	-	-	-	-	1	A1
KU550675	A	-	A	-	-	A	-	-	C	-	-	-	C	G	G	-	C	-	-	-	13	A2
			G	K		T	G										E	K	P			
	T		1	1		2	2										3	4	4			
aa mutation	5	-	3	3	-	6	6	-	-	-	-	-	-	-	-	-	8	5	8	-	124	
	6		3	5		6	8										5	3	4			
	N		S	R		K	E										D	T	H			
second struture	-	-	-	-	-	-	-	-	-	-	H	-	-	H	H	-	H	-	-	-		

Note: M12732 was used as reference. The nucleotides conserved with respect to the reference sequence are marked with a dash(-), whereas a variation position was indicated by a letter. Predicted amino acid changes were also shown. The “S” in the last row of the table means Sheet, the “H” means Helix

### Gene polymorphism of HPV-33 *L2*

Compared with the HPV-33 reference sequence (GenBank: M12732.1), the nucleotide variation rate of HPV-33 *L2* was 100.0 % (101/101) in the 101 HPV-33 *L2* sequences studied. We identified 12 single nucleotide changes among the 101 *L2* sequences studied. Specifically, 5/12 (41.7 %) were synonymous mutations and 7/12 (58.3 %) were non-synonymous mutations. The detected mutations are summarized in Table 3. The maximum-likelihood phylogenetic tree is represented in Fig. 1b. The secondary structure predicting result of the HPV-33 *L2* was showed in Additional file 1: Figure S1 (B).

**Table 3:** Nucleotide sequence mutation at *L2* of 8 HPV-33 isolates

Sequence pattern	HPV33 L2 Nucleotide sequence												*n*	Sub-lineages
	4	4	4	4	5	5	5	5	5	5	5	5		
	4	6	8	9	0	0	2	2	2	3	3	4		
	3	7	0	0	2	5	2	5	8	1	2	5		
	8	1	9	5	6	1	0	7	7	4	4	7		
M12732	G	T	C	C	A	T	C	G	G	G	A	C		
KU518344	-	-	-	-	-	-	T	-	-	-	-	-	73	A1
KU518345	-	-	-	-	-	-	T	-	-	A	-	-	3	A1
KU518346	-	-	-	-	-	-	T	-	-	-	-	T	1	A1
KU518347	-	-	-	-	G		T	-	-	-	-	-	1	A1
KU518348	-	-	-	-	-	A	T	-	-	-	-	-	8	A1
KU518349	A	-	-	-	-	-	T	-	-	-	-	-	6	A1
KU518350	A	-	A	T	-	-	T	-	-	-	-	-	3	A1
KU518351	A	C	-	T	-	-	T	C	A	-	C	-	6	A2
					I	F		D	D	D	N			
	D				2	2		3	3	3	3			
aa mutation	7	-	-	-	7	8	-	5	6	6	7	-	101	
	7				3	1		0	0	9	2			
	N				V	Y		H	N	N	T			
second struture	-	S	-	S	-	-	-	-	-	-	-	-		

Note: M12732 was used as reference. The nucleotides conserved with respect to the reference sequence are marked with a dash(-), whereas a variation position was indicated by a letter. Predicted amino acid changes were also shown. The “S” in the last row of the table means Sheet, the “H” means Helix

### HPV-58 *L1* gene polymorphism

Compared with the HPV-58 reference sequence (GenBank: D90400), the nucleotide variation rate of HPV-58 *L1* was 96.86 % (216/223) in the 223 HPV-58 L1 sequences studied. We identified 32 single nucleotide changes among the 223 sequences studied.

**Fig. 2:**
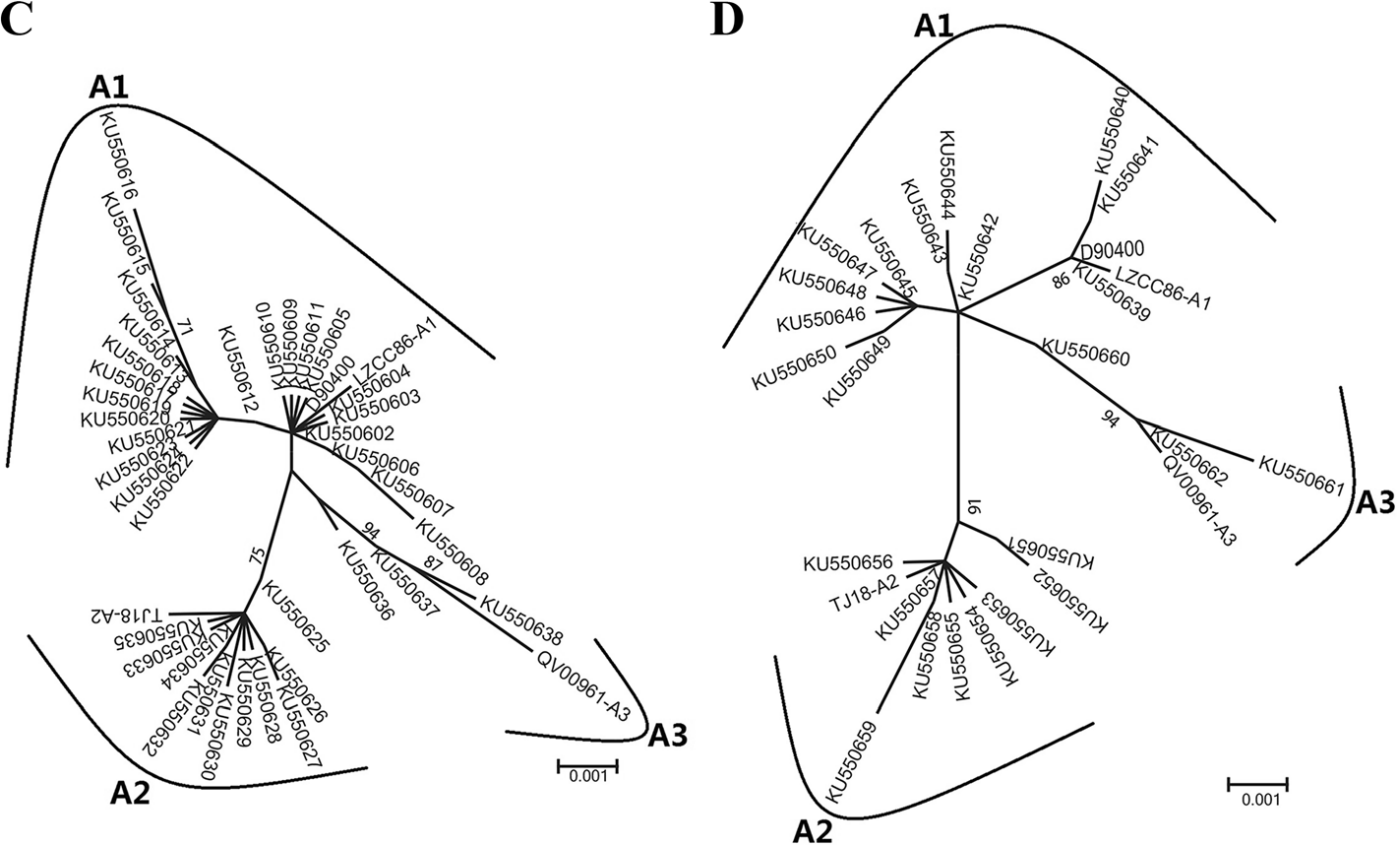
Maximum-likelihood trees of HPV-58 variants. *Note*: Phylogenetic trees based on the Maximum-likelihood method of representing the relationships between variants of HPV-58, (**c**) Tree based on gene *L1*, (D) Tree based on gene *L2*. Sub-lineage classification based on full genomes from a roported research [27], and a representative sequence were chosen from the tree, respectively (LZCC86-A1/EU918765, TJ18-A2/GQ472850, QV00961-A3/HQ537758). Numbers above the branches indicate the bootstrap values that are greater than 70 %

Specifically, 23/32 (71.88 %) were synonymous mutations and 9/32 (28.12 %) were non-synonymous mutations. 3 non-synonymous mutations were observed in sequences encoding the helix, 1 non-synonymous mutation was observed in sequence encoding the sheet. The detected mutations are summarized in Table 4. The maximum-likelihood phylogenetic tree is shown in Fig. 2c. The secondary structure predicting result of the HPV-58 *L1* was showed in Additional file 2: Figure S2 (C).

**Table 4:** Nucleotide sequence mutation at *L1* of 37 HPV-58 isolates

Sequence pattern	HPV58 L1 Nucleotide sequence																																		*n*	Sub-lineages
	5	5	5	5	5	5	5	5	5	5	6	6	6	6	6	6	6	6	6	6	6	6	6	6	6	6	6	6	6	6	6	6	6	6		
	7	7	8	8	8	8	8	8	8	9	0	4	4	4	4	4	4	4	5	5	5	5	5	5	6	6	6	7	7	8	8	8	8	9		
	4	9	0	0	0	2	2	3	7	8	1	0	1	3	3	5	7	8	0	0	1	2	3	6	4	8	9	9	9	2	2	6	8	9		
	7	8	1	7	9	2	8	4	3	4	4	4	6	4	7	8	0	5	0	3	7	1	9	0	1	8	5	7	8	7	9	0	1	9		
D90400	T	C	A	C	A	A	A	C	T	G	A	G	A	T	A	G	T	T	C	A	T	A	A	A	G	C	A	G	A	C	A	A	A	A		
KU550602	-	-	-	-	-	-	-	-	-	-	-	-	-	-	-	-	-	-	-	-	-	-	-	-	-	-	-	-	-	-	-	-	-	-	7	A1
KU550603	-	-	-	A	-	-	-	-	-	-	-	-	-	-	-	-	-	-	-	-	-	-	-	-	-	-	-	-	-	-	-	-	-	-	13	A1
KU550604	-	-	-	-	-	-	-	-	-	-	-	-	-	-	G	-	-	-	-	-	-	-	-	-	-	-	-	-	-	-	-	-	-	-	4	A1
KU550605	-	-	-	-	-	-	-	-	-	-	-	-	-	-	-	-	-	-	-	G	-	-	-	-	-	-	-	-	-	-	-	-	-	-	2	A1
KU550606	-	-	-	-	-	-	-	-	-	-	-	-	-	-	-	-	-	-	-	-	-	-	-	-	-	-	-	-	-	-	-	G	-	-	31	A1
KU550607	-	-	-	-	-	-	-	-	-	-	-	-	-	-	-	-	-	-	-	-	-	-	-	-	-	-	C	-	-	-	-	G	-	-	1	A1
KU550608	-	-	-	-	-	-	-	-	-	-	-	-	-	-	-	-	-	-	-	-	-	-	-	-	-	-	C	A	-	-	-	-	-	-	1	A1
KU550609	-	-	-	-	-	-	G	-	-	-	-	-	-	-	-	-	-	-	-	-	-	-	-	-	-	-	-	-	-	-	-	-	-	-	1	A1
KU550610	-	-	-	-	-	-	-	-	-	-	-	-	-	-	-	A	-	-	-	-	-	-	-	-	-	-	-	-	-	-	-	-	-	-	4	A1
KU550611	-	-	-	-	-	-	-	-	-	-	-	A	-	-	-	-	-	-	-	-	-	-	-	-	-	-	-	-	-	-	-	-	-	-	5	A1
KU550612	-	-	-	-	-	-	-	-	-	-	-	-	-	-	-	-	-	-	-	-	-	-	-	G	-	-	-	-	-	-	-	-	-	-	4	A1
KU550613	-	-	-	-	-	-	-	-	-	-	C	-	-	-	-	-	-	-	-	-	-	-	-	G	-	A	-	-	-	-	-	-	-	-	1	A1
KU550614	C	-	-	-	-	-	-	-	-	-	C	-	-	-	-	-	-	-	-	-	-	-	-	G	-	A	-	-	-	-	-	-	-	-	2	A1
KU550615	-	-	-	-	-	-	G	-	-	-	C	-	-	-	-	-	-	-	-	-	A	-	-	-	-	A	-	-	-	-	-	-	-	-	1	A1
KU550616	-	T	-	-	C	-	-	A	-	-	C	-	-	-	-	-	-	-	-	-	A	-	-	-	-	A	-	-	-	-	-	-	-	-	3	A1
KU550617	-	-	-	-	-	-	-	-	-	-	-	A	-	-	-	-	-	-	-	-	-	-	-	G	-	A	-	-	-	-	-	-	-	-	2	A1
KU550618	-	-	-	-	-	-	-	-	-	-	-	-	-	-	-	A	-	-	-	-	-	-	-	G	-	A	-	-	-	-	-	-	-	-	3	A1
KU550619	-	-	-	-	-	-	-	-	-	-	-	-	-	-	-	-	-	-	-	-	-	-	-	G	-	A	-	-	-	A	-	-	-	-	2	A1
KU550620	-	-	-	-	-	G	-	-	-	-	-	-	-	-	-	-	-	-	-	-	-	-	-	G	-	A	-	-	-	-	-	-	-	-	1	A1
KU550621	-	-	-	-	-	-	-	-	-	-	-	-	-	-	-	-	-	-	-	-	-	-	-	G	-	A	-	-	-	-	-	-	-	-	36	A1
KU550622	-	-	-	-	-	-	-	-	-	-	-	-	-	-	-	-	C	-	-	-	-	-	-	G	-	A	-	-	-	-	-	-	-	-	6	A1
KU550623	-	-	-	-	-	-	-	-	-	A	-	-	-	-	-	-	-	-	-	-	-	-	-	G	-	A	-	-	-	-	-	-	-	-	3	A1
KU550624	-	-	-	-	-	-	-	-	-	-	-	-	-	-	-	-	-	-	-	-	-	-	-	G	-	A	-	-	-	-	C	-	-	-	2	A1
KU550625	-	-	-	-	-	-	-	-	-	-	-	-	G	C	-	-	-	-	-	-	-	-	G	-	A	-	-	-	-	-	-	-	-	-	1	A2
KU550626	-	-	-	-	-	-	-	-	-	-	C	-	G	C	-	-	-	C	-	-	-	-	G	-	A	-	-	-	-	-	-	-	-	-	6	A2
KU550627	-	-	-	-	-	-	-	-	-	-	-	-	G	C	-	-	-	C	-	-	-	-	G	-	A	-	-	-	-	-	-	-	-	-	1	A2
KU550628	-	-	-	-	-	-	-	-	-	-	C	-	G	C	-	-	-	-	-	-	-	-	G	G	A	-	-	-	-	-	-	-	-	-	2	A2
KU550629	-	-	-	-	-	-	-	-	-	-	C	-	G	C	-	A	-	-	-	-	-	-	G	-	A	-	-	-	-	-	-	-	-	-	4	A2
KU550630	-	-	-	-	-	-	G	-	-	-	C	-	G	C	-	-	-	-	-	-	-	G	G	-	A	-	-	-	-	-	-	-	-	-	4	A2
KU550631	-	-	-	-	-	-	-	-	-	-	C	-	G	C	-	-	-	-	-	G	-	-	G	-	A	-	-	-	-	-	-	-	-	-	3	A2
KU550632	-	-	-	-	-	-	-	-	G	-	C	-	G	C	-	-	-	-	-	G	-	-	G	-	A	-	-	-	-	-	-	-	-	-	2	A2
KU550633	-	-	G	-	-	-	-	-	-	-	C	-	G	C	-	-	-	-	-	-	-	-	G	-	A	-	-	-	-	-	-	-	-	-	5	A2
KU550634	-	-	-	-	-	-	-	-	-	-	C	-	G	C	-	-	-	-	-	-	-	-	G	-	A	-	-	-	-	-	-	-	-	-	28	A2
KU550635	-	-	-	-	-	-	-	-	-	-	C	-	G	C	-	-	-	-	-	-	-	-	G	-	A	-	-	-	-	-	-	-	-	C	9	A2
KU550636	-	-	-	-	-	G	-	-	-	-	-	-	-	-	-	-	-	-	-	-	-	-	G	-	-	-	-	-	-	A	-	-	-	-	5	A3
KU550637	C	-	-	-	-	-	-	-	-	-	C	-	-	-	-	-	-	-	-	-	-	-	G	-	-	-	-	-	-	A	-	-	-	-	8	A3
KU550638	C	-	-	-	-	-	-	-	-	-	C	-	-	-	-	-	-	-	T	-	-	-	G	-	-	-	-	-	G	A	-	-	G	-	10	A3
											L										F		I			T	E		I		N			K		
					N						1										3		3			3	3		4		4			4		
aa mutation	-	-	-	-	8	-	-	-	-	-	5	-	-	-	-	-	-	-	-	-	1	-	2	-	-	7	7	-	1	-	2	-	-	7	223	
					2						0										8		5			5	7		2		2			9		
					T						F										Y		M			N	D		V		T			Q		
second structure	S	S	S	-	-	-	S	-	S	-	-	H	H	-	-	-	-	-	-	-	H	S	S	-	S	-	-	H	H	-	-	-	-	-		

Note: D90400 was used as reference. The nucleotides conserved with respect to the reference sequence are marked with a dash(-), whereas a variation position was indicated by a letter. Predicted amino acid changes were also shown. The “S” in the last row of the table means Sheet, the “H” means Helix

### HPV-58 *L2* gene polymorphism

Compared with the HPV-58 reference sequence (GenBank: D90400), the nucleotide variation rate of HPV-58 *L2* was 68.55 % (168/201) in the 124 HPV-58 *L2* sequences studied. We identified 26 single nucleotide changes among the 201 *L2* sequences studied. Specifically, 17/26 (65.38 %) were synonymous and 9/26 (34.62 %) were non-synonymous mutations. 3 non-synonymous mutations were observed in sequences encoding the sheet. The detected mutations are summarized in Table 5. The maximum-likelihood phylogenetic tree is displayed in Fig. 2d. The secondary structure predicting result of the HPV-58 *L2* was showed in Additional file 2: Figure S2 (D).

**Table 5:** Nucleotide sequence mutation at *L2* of 24 HPV-58 isolates

Sequence pattern	HPV58 L2 Nucleotide sequence																									*n*	Sub-lineages
	4	4	4	4	4	4	4	4	4	4	5	5	5	5	5	5	5	5	5	5	5	5	5	5	5		
	4	4	5	5	5	6	6	6	9	9	0	0	0	1	1	2	2	3	3	4	4	4	4	5	5		
	5	7	4	5	7	0	2	2	3	4	0	0	1	1	4	0	6	2	9	3	6	8	8	4	7		
	2	0	9	8	0	9	1	7	5	5	0	0	4	9	3	6	6	6	5	7	7	2	9	0	9		
D90400	G	G	T	A	G	A	A	T	A	A	G	G	A	A	T	A	G	A	G	A	A	A	G	A	A		
KU550639	-	-	-	-	-	-	-	-	-	-	-	-	-	-	-	-	-	-	-	-	-	-	-	-	-	33	A1
KU550640	-	-	-	-	-	-	-	C	-	-	-	-	-	-	-	G	-	-	-	-	-	-	-	-	-	4	A1
KU550641	-	-	-	-	-	-	-	-	-	-	-	-	-	-	-	G	-	-	-	-	-	-	-	-	-	18	A1
KU550642	-	-	-	-	-	-	-	C	-	-	-	-	-	-	-	-	-	-	C	-	-	-	A	-	-	5	A1
KU550643	-	-	-	-	-	-	-	C	-	-	-	-	-	-	-	-	-	T	C	-	-	-	A	-	-	1	A1
KU550644	-	-	-	-	-	-	C	C	-	-	-	-	-	-	-	-	-	T	C	-	-	-	A	-	-	1	A1
KU550645	-	-	-	-	-	-	C	C	-	-	-	-	-	-	-	-	-	-	C	-	-	-	A	-	-	36	A1
KU550646	-	A	-	-	-	-	C	C	-	-	-	-	-	-	-	-	-	-	C	-	-	-	A	-	-	6	A1
KU550647	-	-	-	-	-	-	C	C	-	-	-	C	-	-	-	-	-	-	C	-	-	-	A	-	-	1	A1
KU550648	A	-	-	-	-	-	C	C	-	-	-	-	-	-	-	-	-	-	C	-	-	-	A	-	-	4	A1
KU550649	-	-	-	G	-	-	C	C	-	-	-	-	-	-	-	-	-	-	C	-	-	-	A	-	-	1	A1
KU550650	-	-	G	G	-	-	C	C	-	-	-	-	-	-	-	-	-	-	C	-	-	-	A	-	-	4	A1
KU550651	-	-	-	-	-	-	-	C	-	G	-	-	G	G	G	-	A	-	C	-	C	-	A	-	-	10	A2
KU550652	-	-	-	-	-	-	C	C	-	G	-	-	G	G	G	-	A	-	C	-	C	-	A	-	-	2	A2
KU550653	-	-	-	-	-	-	C	C	-	G	-	-	G	G	G	-	A	-	C	-	-	-	A	C	-	1	A2
KU550654	-	-	-	-	-	G	-	C	-	G	-	-	G	G	G	-	A	-	C	-	-	-	A	C	-	2	A2
KU550655	-	-	-	-	-	-	-	C	-	G	-	-	G	G	G	-	A	C	C	-	-	-	A	C	-	8	A2
KU550656	-	-	-	-	-	-	-	C	-	G	-	-	G	G	G	-	A	-	C	-	-	T	A	C	-	7	A2
KU550657	-	-	-	-	-	-	-	C	-	G	-	-	G	G	G	-	A	-	C	-	-	-	A	C	-	25	A2
KU550658	-	-	-	-	-	-	-	C	-	G	-	-	G	G	G	-	A	-	C	G	-	-	A	C	-	6	A2
KU550659	-	-	-	-	-	-	-	C	-	G	T	-	G	G	G	-	A	-	C	G	-	-	A	C	-	1	A2
KU550660	-	-	-	-	A	G	-	C	-	-	-	-	-	-	-	-	-	-	C	-	-	-	A	-	-	1	A3
KU550661	-	-	-	-	-	-	-	-	C	-	-	-	-	-	-	-	A	-	C	-	-	-	A	-	C	2	A3
KU550662	-	-	-	-	A	G	-	C	C	-	-	-	-	-	-	-	A	-	C	-	-	-	A	-	C	22	A3
									N		D	D											A	T	M		
	G	S							2		2	2											4	4	4		
aa mutation	7	7	-	-	-	-	-	-	3	-	5	5	-	-	-	-	-	-	-	-	-	-	1	3	4	201	
	0	6							1		3	3											6	3	6		
	E	N							T		Y	H											T	P	L		
second structure	-	-	-	-	-	-	-	-	-	S	S	S	-	S	S	-	-	-	S	-	-	-	S	-	-		

Note: D90400 was used as reference. The nucleotides conserved with respect to the reference sequence are marked with a dash(-), whereas a variation position was indicated by a letter. Predicted amino acid changes were also shown. The “S” in the last row of the table means Sheet, the “H” means Helix

### Selective pressure analysis

The variable dN/dS rate ratios were tested among the various lineages using the PAML4.8 [10]. HPV-33 *L1*-positive selection was seen with T56N, G133S, K135R, T226K, G268E and E385D. HPV-33 L2-positive selection was seen in D77N, D350H, D360N and N372T. The HPV-58 *L1*-positive selection included L150F, F318Y, I325M, T375N and E377D. The HPV-58 *L2*-positive lineages included N231T, A416T and M446L. Results of the selective pressure analysis of HPV-58 and HPV-33 *L1* and *L2* genes (*P*-value, 0.1) are summarized in Tables 6, 7, 8 and 9.

**Table 6:** Site-specific tests for positive selection on HPV-33 L1

Models	InL	Estimates of parameters	2Δl	Positively selected sites
M7	−2415.234	*p* = 0.005 q = 0.048		NA
M8	−2395.143	*p*0 = 0.982 *p* = 0.114 q =6.861 (*p*1 = 0.018) ω = 16.384	40.183 *p* < 0.01	56 T**, 133G**, 135 K*, 266 T**, 268G**, 385E*

**Table 7:** Site-specific tests for positive selection on HPV-33 L2

Models	InL	Estimates of parameters	2Δl	Positively selected sites
M7	−2185.718	*p* = 0.005 q = 0.020		NA
M8	−2174.779	*p*0 = 0.985 *p* = 0.551 q = 5.040 (*p*1 = 0.015) ω = 20.381	21.878 *p* < 0.01	77D**, 350D**, 360D*, 372 N**

**Table 8:** Site-specific tests for positive selection on HPV-58 L1

Models	InL	Estimates of parameters	2Δl	Positively selected sites
M7	−2849.987	*p* = 0.005 q = 0.048		NA
M8	−2821.953	*p*0 = 0.995 *p* = 0.006 q = 0.096 (*p*1 = 0.005) ω = 22.821	56.068 *p* < 0.01	150 L**, 318 F, 325I**, 375 T**, 377E

**Table 9:** Site-specific tests for positive selection on HPV-58 L2

Models	InL	Estimates of parameters	2Δl	Positively selected sites
M7	−2432.604	*p* = 0.005 q = 0.048		NA
M8	−2424.886	*p*0 = 0.978 *p* = 2.63 q = 99.000 (*p*1 = 0.022) ω = 5.255	15.436 *p* < 0.01	231 N*,416A*,446 M*

Note: Tables 6, 7, 8 and 9: ln L, the log-likelihood difference between the two models; 2Δl, twice the log-likelihood difference between the two models; the positively selected sites were identified with posterior probability ≥ 0.9 using Bayes empirical Bayes (BEB) approach ne asterisk indicates posterior probability ≥ 0.95, and two asterisks indicate posterior probability ≥ 0.99. NA means not allowed. NS means the sites under positive selection but not reaching the significance level of 0.9

### Homology models analysis

**Fig. 3:**
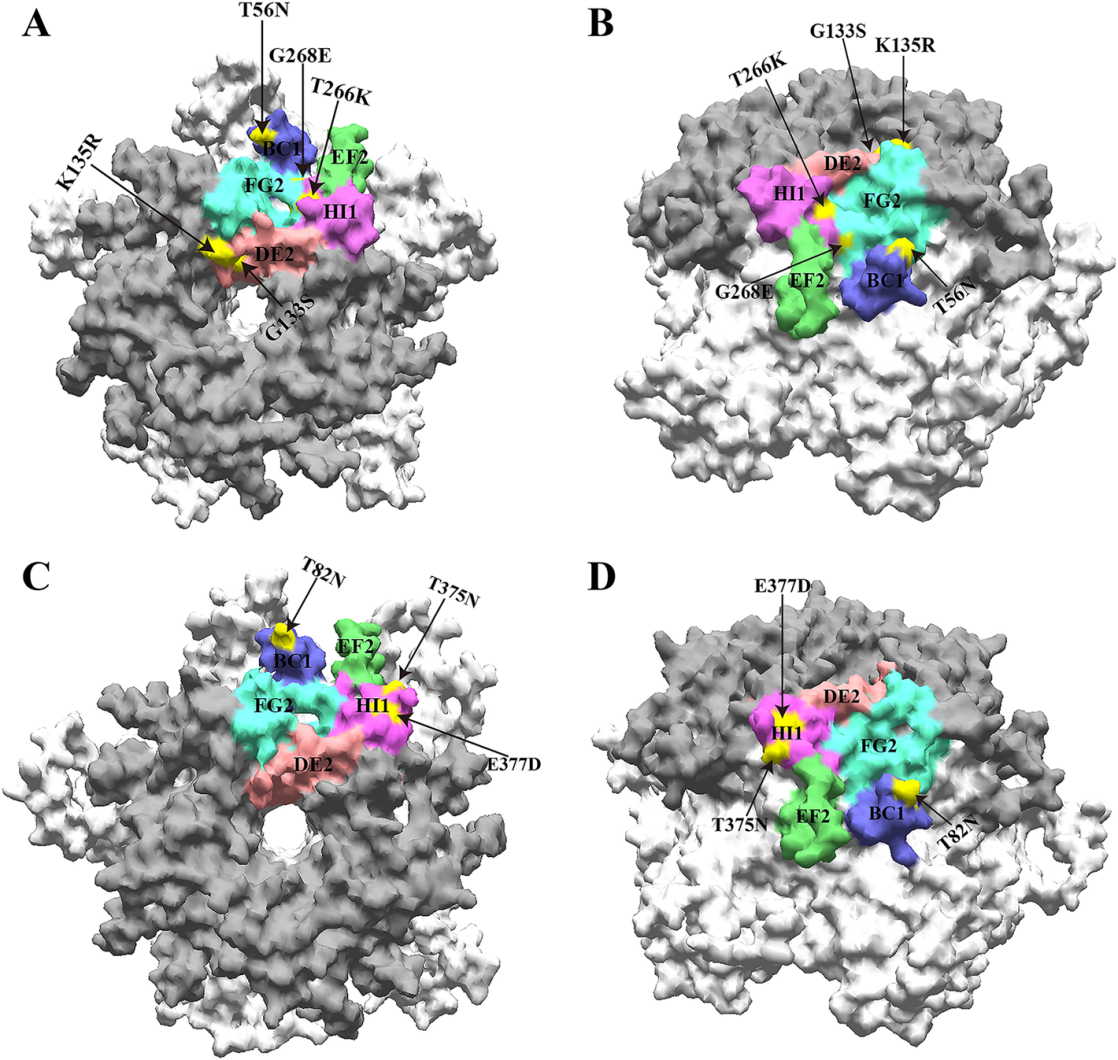
Homology modeling of HPV-33 and HPV-58 variants. *Note*: Homology models based on the HPV-16 L1 pentamer crystal (PDB code: 2R5H). **a** top and (**b**) side surface-filled views shown for HPV-33 and (**c**) top and (**d**) side surface-filled views shown for HPV-58 with external loops indicated by dark grey shading. While all five monomers of the capsomer are pictured, adjacent external loops are indicated (purple, HI1; blue, BC1; red, DE2; cyan FG2; green, EF2), and amino acid mutation is shown with black arrows and marked with yellow color in one of the five copies of a residue

HPV-16, 33, and 58 are known to be closely related and belong to the α-9 species. The whole protein sequences of HPV-16, 33, and 58 *L1* were aligned (Additional file 3: Figure S3) and the BC-loop, DE-loop, EF-loop, FG-loop, and HI-loop of HPV-33 and HPV-58 *L1* were predicted in the present study (Fig. 3). G4438A (T56N) of HPV-33 L1 and A5809C (N82T) of HPV-58 L1 were found in the BC-loop; G5990A (G133S) and G5997A (K135R) of HPV-33 were found in the DE-loop; C6390A (T266K) and G6396A (G268E) of HPV-33 L1 were found in the FG-loop; and C6688A (T375N) and A6695C (E377D) of HPV-58 L1 were found in the HI-loop.

## Discussion

In our previous study of 10682 patients, 3370 (31.5 %) were positive for HPV infection. High-risk subtypes were as follows: HPV16 (*n* = 791;27.1 %), HPV58 (*n* = 476; 16.3 %), HPV33 (*n* = 273; 9.4 %), HPV52 (*n* = 244; 8.4 %), HPV18 (*n* = 201; 6.9 %), HPV56 (*n* = 191; 6.6 %), HPV66 (*n* = 134; 4.6 %), HPV31 (*n* = 123; 4.2 %), HPV59 (*n* = 93; 3.2 %), HPV68 (*n* = 78; 2.7 %), HPV51 (*n* = 72; 2.5 %), HPV35 (*n* = 63, 2.2 %), HPV53 (*n* = 58; 2.0 %), HPV45 (*n* = 34; 1.2 %), HPV73 (*n* = 29; 1.0 %), HPV39 (*n* = 30; 1.0 %), HPV83 (*n* = 20; 0.7 %), and MM4 (*n* = 5;0.2 %). Low-risk genotypes included HPV6 (*n* = 703; 40.8 %), HPV11 (*n* = 505; 29.3 %), HPV43 (*n* = 403; 23.4 %), HPV42 (*n* = 112; 6.5 %), and HPV44 (*n* = 1; 0.1 %) [7]. Furthermore, the detection rates of HPV-16 and 18 had decreased and that of HPV-58 and HPV-33 had increased over a 6-year period [7]. In addition to the analysis of the treatment and prevention in China, we also obtained data related to the DNA of HPV-33 and HPV-58.

Chen et al showed that HPV-33 manifested two viral lineages (A and B). The HPV-58 was classified into four variant lineages (A, B, C and D) [27]. In the 124 HPV-33 *L1*, our study samples matched with clade A, 111 samples in A1, and 13 samples in A2. All the 101 HPV-33 *L2* study samples matched clade A, 88 samples in A1, and 13 samples in A2. All the 223 HPV-58 *L1* study samples matched clade A, 135 samples in A1, 65 samples in A2, and 23 samples in A3. In case of 201 HPV-58 *L2*, all our study samples matched clade A, 114 samples in A1, 62 samples in A2, and 25 samples in A3. The sequence patterns of HPV-33 *L1* and *L2* were 13 and 8, and for HPV-58, they were 37 and 24, respectively.

We demonstrated that HPV-58 *L1* and *L2* variation frequencies were higher than those of HPV-33 *L1* and *L2*. Among these variations, C5807A, A5822G, G5984A, A6437G, T6470C, T6485C and A6695C (E377D, which is a positive selection variation) represented novel HPV-58 *L1* mutations, which were found until now only in Sichuan, China [15, 17, 28–32]. In HPV-58 *L2*, mutations other than A4621C and A5206G, were newly reported [15]. In HPV-33 *L1*, T5960C, A5997G (K135R), T6385G, G6396A (G268E), T6463C, G6520A, T6613C, A6694G, A6951C (K453T), C7044A (P484H) and G7063A were reported for the first time, these newly reported mutations were only found in China in reports related to HPV-33 *L1* [30, 33, 34]. We reported the HPV-33 L2 mutations for the first time.

The most common non-synonymous mutations of HPV-33 *L1* included T266K (18/124), T56N (13/124), G133S (13/124) and E385D (13/124). The most common non-synonymous mutations of HPV-33 *L2* were D77N (15/101), F281Y (8/101), D350H (6/101), D360N (6/101), and N372T (6/101). The most common non-synonymous mutations of HPV-58 *L1* comprised L150F (88/223), I135M (88/223), and T375N (62/223). The most common non-synonymous mutations of HPV-58 *L2* were A416T (146/201), N231T (24/201), and M446L (24/201). Results of the selective pressure analysis suggest that most positive selection mutations of HPV-33 and HPV-58 *L1*/*L2* were common non-synonymous mutations, indicating that the positive selection variations beneficial for HPV-33 and HPV-58 adapted to their environments widely. The other variations in the positive selection mutations were uncommon. However, with the inheritance of the virus, positive variations may become increasingly common and gain importance in the future. The most common synonymous mutations of HPV-33 *L1* were A6664G (18/124), T6613C (14/124), T6478C (13/124) and A6673G (13/124). The most common synonymous mutations of the HPV-33 *L2* included C5220T (101/101) and C4905T (9/101). The most common synonymous mutations of HPV-58 *L1* were A6416G (65/223), T6434C (65/223), G6641A (65/223), and A6560G (64/223); and those of the HPV-58 *L2* were T4627C (148/201), G5395C (146/201), and G5266A (86/201). Due to the high diagnostic value of *L1*, and its variability [35], *L1* is often selected as a clinical diagnostic target. We considered the most sites in common mutations to design clinical diagnostic probes targeting HPV *L1* and *L2* genes. The intratypic variations observed in *L1* and *L2* enabled the analysis of known and novel HPV subtypes [36, 37]. In our study, we observed that the sequence patterns and single nucleotide changes of HPV-33 *L2* were less frequent than those of HPV-33 *L1*. The sequence and single nucleotide changes of HPV-58 *L2* were less frequent than those of HPV-58 *L1*, while those of HPV-33 and HPV-58 *L2* were more conserved than those of *L1*, suggesting that HPV-33 and HPV-58 *L2* were better candidates as clinical diagnostic targets compared with HPV-33 and HPV-58 *L1*.

Nearly all conformational epitopes are located on one or more of the outwardly facing surface-exposed loops of BC, DE, EF, FG, and HI [38]. Sites 54, 55, 135–139, 141–143, 181, 182, 184, 267, 269, 270, 273, 278, 280, 282–287, 348, 354, 358, and 361 were previously reported to be important for the Fab interaction [38]. Although we found no mutations at these Fab interaction sites, we did find several mutations (T56N, K135R, T266K, and G268E of HPV-33 *L1*; N82T of HPV-58 *L1*) next to these sites. We believe mutations occurred on the outwardly facing surface-exposed loops deserve research attention and should be studied for vaccine design targeting HPV-33 and 58 *L1*.

Amino acid residues 69–81 and 108–120 of L2 protein are highly conserved and contain cross-reacting epitopes that play an important role in inducing neutralizing antibodies [14]. G4438A (D77N) was discovered at residues 69–81 and 108–120 of HPV-33 *L2*. G4452A (G70E) and G4470A (S76N) were identified at residues 69–81 and 108–120 of HPV-58 *L2*. Amino acid residues 33–52, 73–84, 89–100, and 121–140 of *L2* contain non-neutralizing antibody epitopes [14]. G4438A (D77N) of HPV-33 *L2* and The G4470A (S76N) of HPV-58 *L2* were discovered at residues 73–84. These mutations must be considered during vaccine design targeting HPV-33 and 58 *L2*.

This is the first study examining the role of *L1/L2* proteins of HPV-58 variants in Sichuan and that of the *L1/L2* proteins of HPV-33 in China. Because of limitations related to sample size, sample copies, and sequencing technology, the present study may have had a sampling bias against integrated HPV genomes. The data presented in this study have significant implications for the understanding of intrinsic geographical and biological differences in HPV-33 and HPV-58 *L1/L2*, as well as contribute to the design of clinical diagnostic probes and second-generation polyvalent vaccine based on HPV-33 and HPV-58 *L1/L2*.

## Conclusions

Mutations in HPV *L1* and *L2* may alter the virulence of variants, and also define altered epitopes in vaccine design. The reference sequences of HPV-33 and 58 only represent minor sequence patterns of HPV-33 and 58 *L1/L2*. Further, the distribution of HPV-33 and 58 *L1/L2* variations in Sichuan has its own peculiarities. Therefore, clinical diagnostic probes and second-generation polyvalent vaccines should be designed on the basis of the unique sequence of HPV-33 and 58 *L1/L2* variations in Sichuan, whereby the accuracy of clinical detection and the protective efficiency of vaccines can be improved.

## Additional files

Additional file 1: Figure S1. Predicted of HPV-33 L1 and L2 proteins Secondary Structure by PSIPred. Note: A) Secondary structure within the reference sequence of HPV-33 L1 protein, B) Secondary structure within the reference sequence of HPV-33 L2 protein. Black arrow indicates corresponding mutation is a non-synonymous mutation. (TIF 948 kb)
Additional file 2: Figure S2. Predicted of HPV-58 L1 and L2 proteins secondary structure by PSIPred. Note: C) Secondary structure within the reference sequence of HPV-58 L1 protein, D) Secondary structure within the reference sequence of HPV-58 L2 protein. Black arrow indicates corresponding mutation is a non-synonymous mutation. (TIF 922 kb)
Additional file 3: Figure S3. Sequence alignment of L1 from the HPV types HPV16, HPV33, and HPV58. Note: The residues conserved across four HPV types are shown in capital letters, whereas the nonconserved residues are given in lowercase letters. The five loops displayed on the surface of the virus particle are marked and labeled. (TIF 1448 kb)

## Abbreviations

BEB: Bayes empirical Bayes
Fab: Antigen-binding fragment
HPV: Human papillomavirus
MEGA: Molecular Evolutionary Genetics Analysis Version
PAML: Phylogenetic Analyses by Maximun Likelihood
VLPs: Virus-like particles
