# Supplementary figures and images for: L1 and L2 gene polymorphisms in HPV-58 and HPV-33: implications for vaccine design and diagnosis
Source: Virol J. 2016 Oct 7;13:167. doi: 10.1186/s12985-016-0629-9 (PMC5055703; doi:10.1186/s12985-016-0629-9)

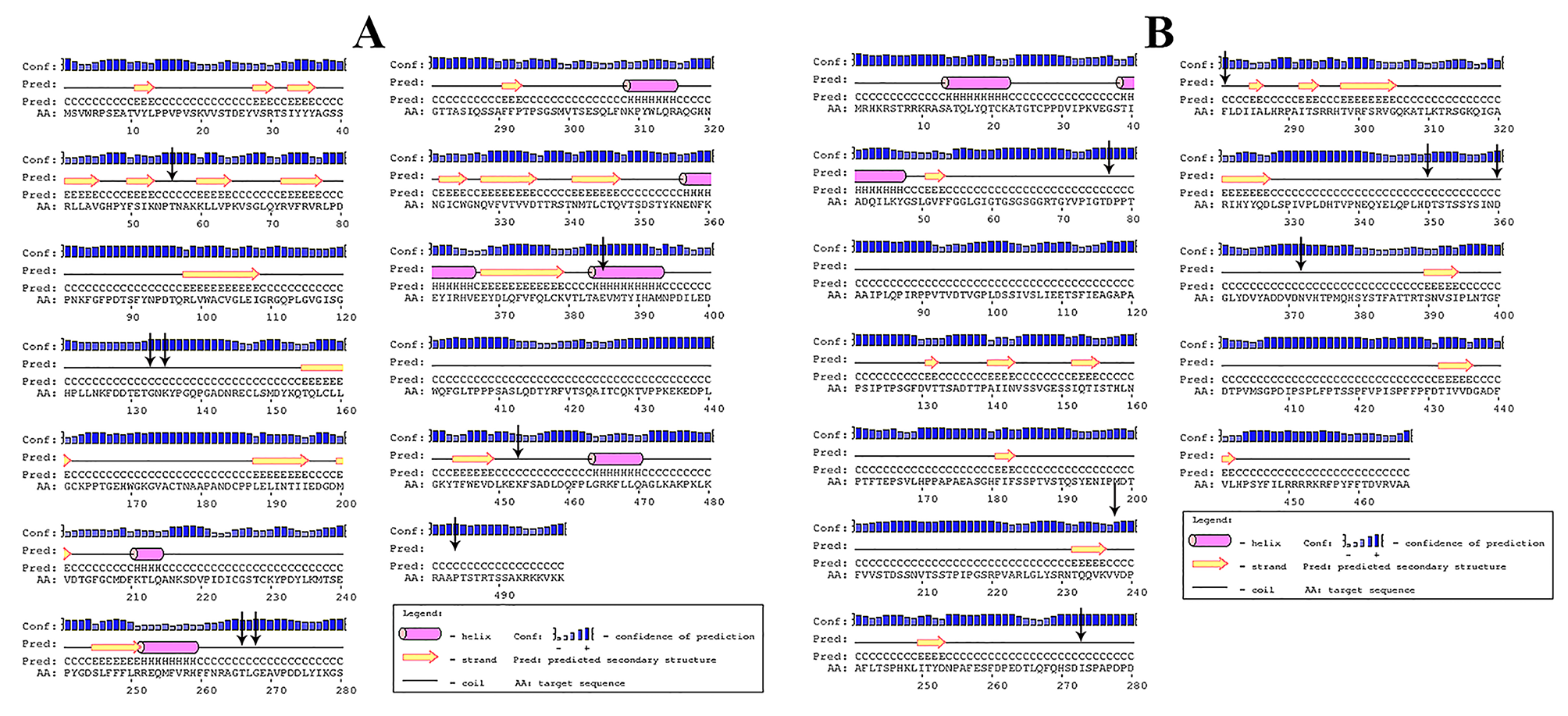

Supplement: Additional file 1: Figure S1. — Predicted of HPV-33 L1 and L2 proteins Secondary Structure by PSIPred. Note: A) Secondary structure within the reference sequence of HPV-33 L1 protein, B) Secondary structure within the reference sequence of HPV-33 L2 protein. Black arrow indicates corresponding mutation is a non-synonymous mutation. (TIF 948 kb) [file 12985_2016_629_MOESM1_ESM.tif]

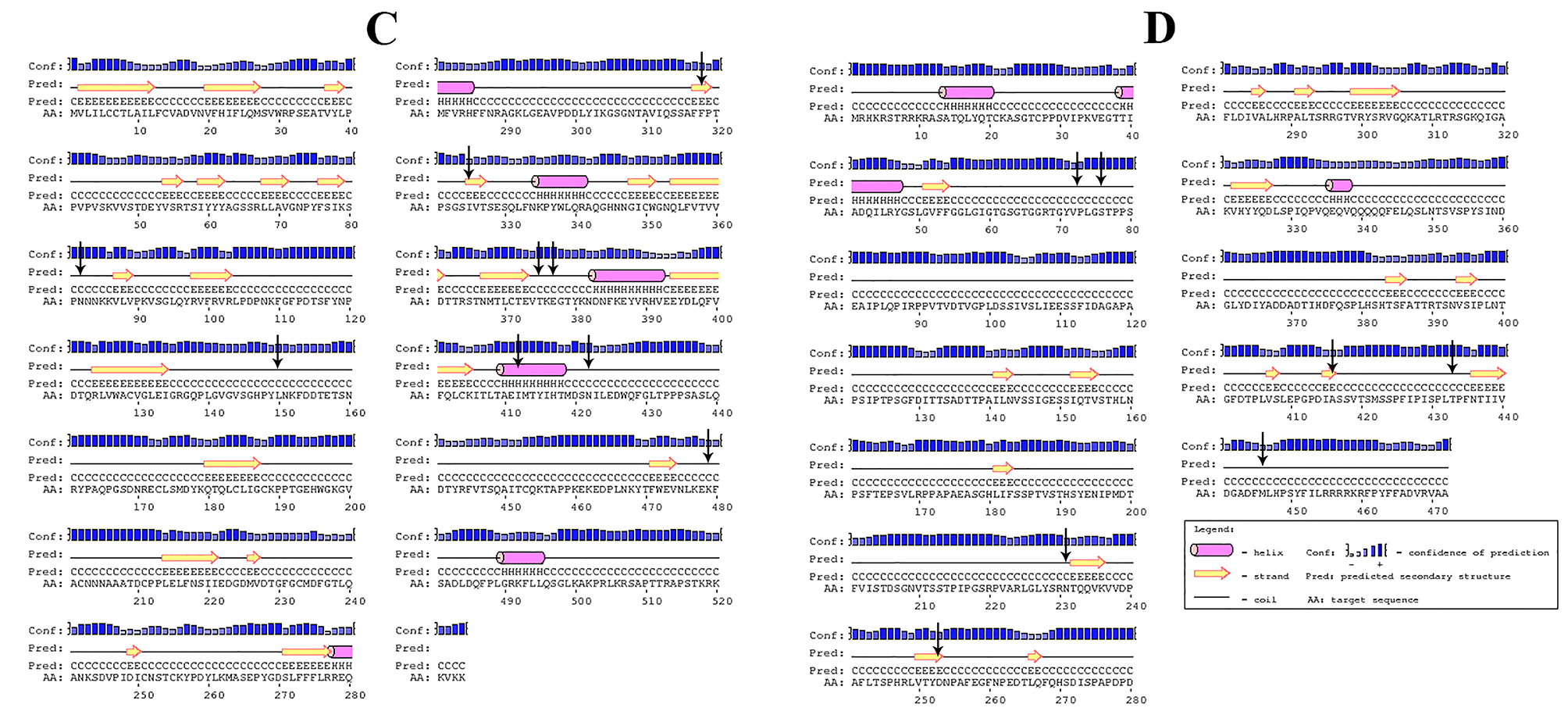

Supplement: Additional file 2: Figure S2. — Predicted of HPV-58 L1 and L2 proteins secondary structure by PSIPred. Note: C) Secondary structure within the reference sequence of HPV-58 L1 protein, D) Secondary structure within the reference sequence of HPV-58 L2 protein. Black arrow indicates corresponding mutation is a non-synonymous mutation. (TIF 922 kb) [file 12985_2016_629_MOESM2_ESM.tif]

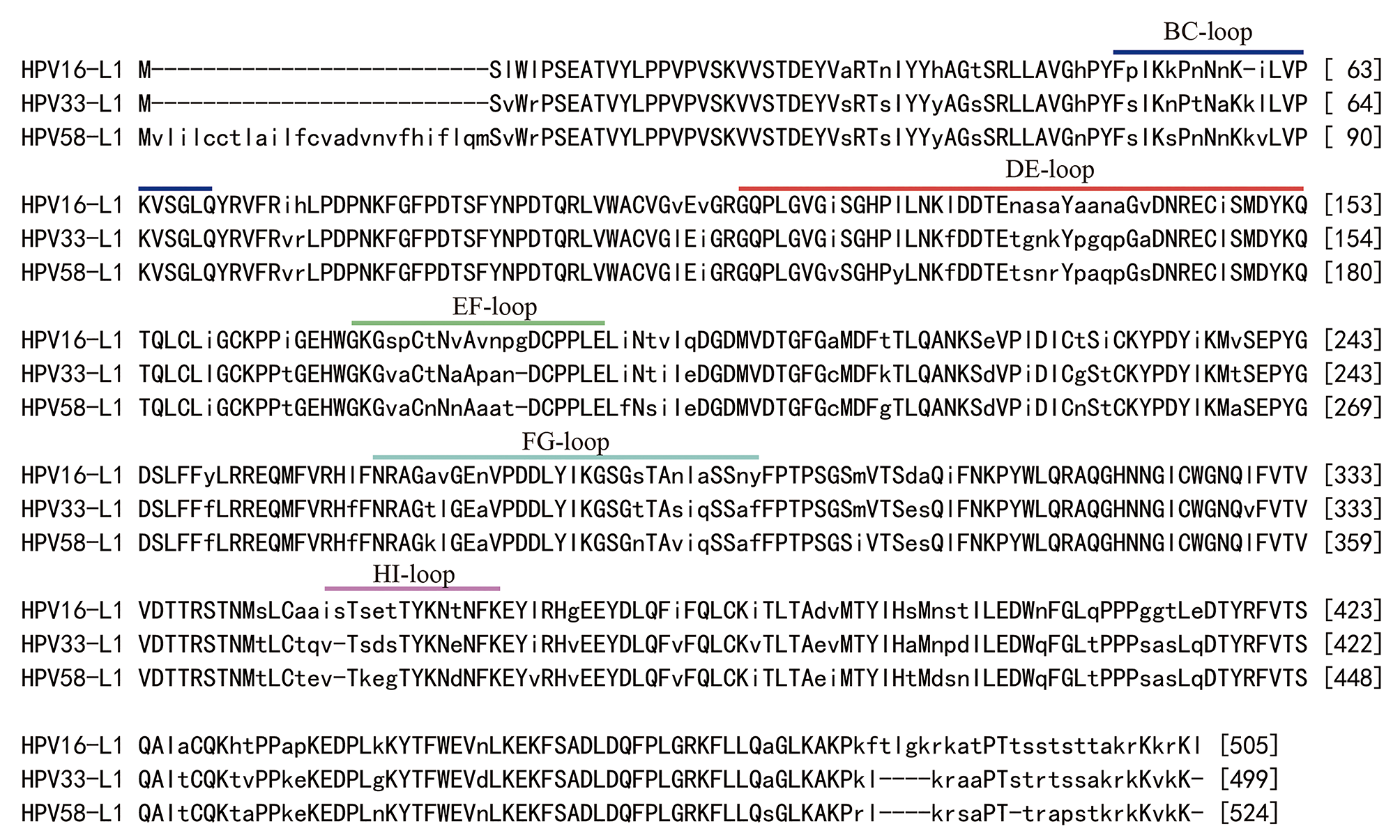

Supplement: Additional file 3: Figure S3. — Sequence alignment of L1 from the HPV types HPV16, HPV33, and HPV58. Note: The residues conserved across four HPV types are shown in capital letters, whereas the nonconserved residues are given in lowercase letters. The five loops displayed on the surface of the virus particle are marked and labeled. (TIF 1448 kb) [file 12985_2016_629_MOESM3_ESM.tif]
